# Single-molecule fluorescence studies on cotranscriptional G-quadruplex formation coupled with R-loop formation

**DOI:** 10.1093/nar/gkaa695

**Published:** 2020-08-18

**Authors:** Gunhyoung Lim, Sungchul Hohng

**Affiliations:** Department of Physics and Astronomy, and Institute of Applied Physics, Seoul National University, Seoul 08826, Republic of Korea; Department of Physics and Astronomy, and Institute of Applied Physics, Seoul National University, Seoul 08826, Republic of Korea

## Abstract

G-quadruplex (GQ) is formed at various regions of DNA, including telomeres of chromosomes and regulatory regions of oncogenes. Since GQ is important in both gene regulation and genome instability, the biological and medical implications of this abnormal DNA structure have been intensively studied. Its formation mechanisms, however, are not clearly understood yet. We report single-molecule fluorescence experiments to monitor the cotranscriptional GQ formation coupled with R-loop formation using T7 RNA polymerase. The GQ is formed very rarely per single-round transcription. R-loop formation precedes and facilitates GQ formation. Once formed, some GQs are extremely stable, resistant even to RNase H treatment, and accumulate in multiple-round transcription conditions. On the other hand, GQ existing in the non-template strand promotes the R-loop formation in the next rounds of transcription. Our study clearly shows the existence of a positive feedback mechanism of GQ and R-loop formations, which may possibly contribute to gene regulation and genome instability.

## INTRODUCTION

G-quadruplex (GQ) is a noncanonical nucleic acid structure consisting of two or more stacks of G-quartets, which are made by Hoogsteen base pairing of four guanines (1). *In vitro*, it is preferentially formed in the presence of potassium ions (2). Development of various experimental techniques that use GQ-specific antibodies or chemical probes (3,4) has confirmed that telomeres, oncogenic promoters and 5′ UTR regions are hotspots for GQ formation (5,6). Since various regulation processes can occur in these regions, positive biological roles of GQ in gene regulation and DNA replication have been expected, and some of them are experimentally confirmed (7–9). On the other hand, GQ may serve as a major obstacle for DNA polymerase and telomerase (10–12), and as a result can cause genome instability (13). Consistently, GQ was reported to be formed more abundantly in cancer cells than normal cells (14).

Since GQ is thermodynamically more favorable than the unstructured single-stranded DNA (ssDNA) in the presence of potassium ions, its formation in the single-stranded region of telomeres can be expected. During DNA replication, GQ can also be formed in long single-stranded regions transiently exposed. It has been reported that GQ preferentially formed in actively transcribed genes (15). Since a long ssDNA is not exposed in normal RNA transcription, it has been an intriguing question how transcription helps GQ formation. Interestingly, R-loops, another noncanonical nucleic acid structure, are preferentially formed in G-rich regions (16). Both GQs and R-loops were found to be formed and coexist only when transcription was allowed (17). Stabilization of GQs using GQ-binding ligands also stabilized the cotranscriptionally formed R-loops (18). On the other hand, removal of R-loops using RNase H treatment destabilized the cotranscriptionally formed GQs (18,19). From these observations, it seems clear that formation of GQs and R-loops during transcription is coupled. However, the coupling mechanisms during transcription still remain unclear. Here, we report single-molecule fluorescence studies that monitor the cotranscriptional formation of GQs and R-loops in real time using T7 RNA polymerase. We clearly show that R-loop is formed first, and GQ later. Once formed, some GQs are extremely stable, resistant to RNase H treatment, and as a result accumulate in multiple-round transcription. On the other hand, GQs formed in the non-template strand help the R-loop formation in the next rounds of transcription.

## MATERIALS AND METHODS

### Preparation of DNA substrates

All DNA oligonucleotides were purchased from Integrated DNA Technologies (Coralville, IA). Double-stranded DNA (dsDNA) substrates were made by annealing and ligating three DNA strands (Supplementary Table S1): two non-template strands, each of which was internally labeled with Cy3 or Cy5, and one template strand labeled with biotin at the 3′-end. The annealing was done in T50 buffer [10 mM Tris–HCl (pH 8.0), 50 mM NaCl] by slowly cooling the mixture from 95 to 4°C. The ligation was done in 1× T4 DNA ligation buffer (New England Biolabs) with T4 DNA Ligase II (New England Biolabs) for 16 h at 16°C. The ligation product was purified with denaturing PAGE gel and re-annealed in the buffer containing 50 mM LiCl and 10 mM Tris–HCl (pH 8.0). When efficient GQ formation was needed in the dsDNA context, the re-annealing reaction was performed in a crowding condition with 50% PEG200 and 100 mM KCl instead of 50 mM LiCl (20).

### Formation of stalled elongation complex

To make elongation complex stalled at +11 position from transcription start site, we mixed the pre-annealed dsDNA (25 nM), ATP (60 μM), GTP (60 μM) and T7 RNAP (80 nM) in the solution containing Tris–HCl (40 mM, pH 8.0), KCl (50 mM), MgCl_2_ (20 mM) and DTT (1 mM), and incubated the mixture for 40 min in a heat block maintained at 37°C.

### Preparation of Alexa488-labeled antibody

The primary antibody S9.6 (catalog no. ENH001) was purchased from Kerafast Inc. The secondary antibody (anti-mouse IgG, catalog no. 715-005-150) was purchased from Jackson ImmunoResearch Inc. The secondary antibody was fluorescently labeled by incubating 1.1 mg/ml secondary antibody and 2 mM Alexa488 maleimide (Thermo Fisher) in PBS buffer containing 100 mM NaHCO_3_ for 1 h on the rotator, and purified twice using a NAP-5 gel filtration column. The labeling efficiency was measured as 94%, indicating that most antibodies were successfully labeled.

### Single-molecule fluorescence experiments

Quartz slides and glass coverslips were cleaned using piranha solution (2:1 mixture of 95% sulfuric acid and 30% hydrogen peroxide), treated with (3-aminopropyl)trimethoxysilane for 30 min, and then coated with a 40:1 mixture of PEG (m-PEG-5000, Laysan Bio) and biotin-PEG (biotin-PEG-5000, Laysan Bio). A microfluidic sample chamber (volume: ∼20 μl) was made by assembling the PEG-coated quartz slide and glass coverslip using a double-sided tape. For real-time buffer exchange during experiments using a motor-driven syringe pump (Fusion 100, Chemyx Inc.), plastic tubing was connected to the ends of the channel. A flow rate of 2000 μl/min was used so that buffer exchange time was comparable to the time resolution of the experiment (0.2 s).

For single-round transcription experiments, we immobilized the stalled elongation complexes on a PEG-coated quartz surface using streptavidin–biotin interaction, and resumed the elongation by injecting rNTP (2 mM for each rNTP) into the channel. For multiple-round transcription experiments, we immobilized dsDNA on a PEG-coated quartz surface, and started transcription by injecting T7 RNA polymerases (8 nM) and rNTPs (2 mM for each rNTP) to the channel. For all other experiments that do not require transcription, only bare DNA was immobilized, and experiments were performed without rNTP and RNA polymerase. All single-molecule experiments were performed in an imaging buffer containing 40 mM Tris–HCl (pH 8.0), 50 mM KCl, 5 mM NaOH, 20 mM MgCl_2_, 1 mM DTT, 2 mM spermidine, ∼3 mM Trolox, 5 mM PCA and 4 U/ml PCD (Oriental Yeast Co.). The sample temperature was maintained at 37°C using a commercial temperature control system (Live Cell Instrument, South Korea) that controls all the temperatures of prism, quartz slide glass, objective lens and injected buffer solution.

Single-molecule images were taken using a home-made total internal reflection fluorescence (TIRF) microscope. To excite Cy3, Cy5 and Alexa488, 532-nm solid-state (Compass 215M-50, Coherent), He–Ne gas 633-nm (Cube 640C, Coherent) and 473-nm solid-state (MBL-III-473, CNI) lasers were used, respectively. Single-molecule fluorescence signals were recorded using a camera (iXon DV897ECS-BV, Andor Technology) with a 200 ms or 1 s binning time. Data were analyzed using home-made programs written with IDL (7.0; ITT) and MATLAB (R2013b; The MathWorks), and commercial programs such as Origin (8.0; OriginLab) and Sigma Plot (8.0; Systat Software).

## RESULTS

### Cotranscriptional formation of G-quadruplex is observed at the single-molecule level

**Figure 1.**
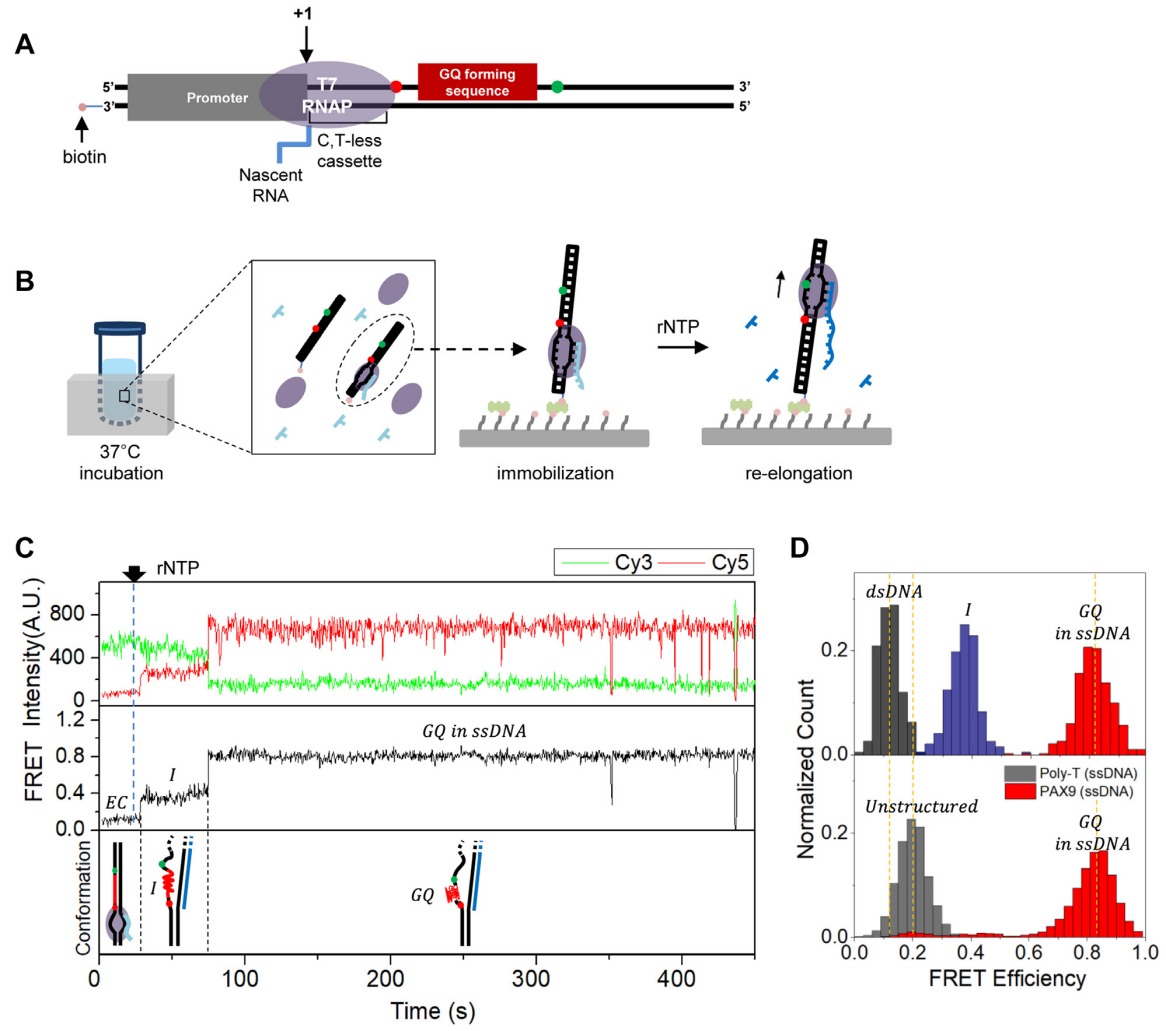
The observation of the cotranscriptional GQ formation under a single-round transcription condition. (**A**) A sample design to make a stalled elongation complex. (**B**) Experimental procedure. After making elongation complexes stalled at +11 position in a tube, they are immobilized on a surface using streptavidin–biotin interaction. The elongation is resumed by injecting rNTP. (**C**) Representative time traces of Cy3 (top, green) and Cy5 (top, red) fluorescence intensities at Cy3 excitation, the corresponding FRET (middle) that show GQ formation and the corresponding conformations of nucleic acids (bottom). The sharp FRET drops around 400 s are due to blinking of dyes. The identities of acronyms in the figure are as follows: EC, stalled elongation complex; I, intermediate state; GQ, G-quadruplex. (**D**) FRET histograms of the low FRET state (top, black), middle FRET state (top, navy) and high FRET state (top, red) are compared with those of unstructured ssDNA consisting of poly-T (bottom, gray) and PAX9 GQ in the ssDNA context (bottom, red).

We designed a DNA substrate that sequentially contains a T7 promoter, C,T-less cassette, GQ forming sequence and 22-nt-long downstream region (Figure 1A). T7 promoter and C,T-less cassette are needed to make stalled elongation complex in a tube. The GQ forming sequence is placed 16 nt downstream of the transcription start site. The length of the dsDNA region downstream of the GQ forming sequence was chosen to be larger than the protected range by T7 RNA polymerase to avoid a possible end effect. To monitor the GQ formation using single-molecule fluorescence resonance energy transfer (FRET) (21), Cy3 (donor) and Cy5 (acceptor) were internally labeled on the non-template strand flanking the GQ forming sequence; the two dyes are >23 nt away, and low to high FRET change is expected upon structural transitions from a duplex form to GQ form. The 3′-end of the template strand (the upstream end) was labeled with biotin for surface immobilization. By incubating the DNA substrate with ATP, GTP and T7 RNA polymerase in a tube, we made elongation complexes in which transcription is stalled with 11-nt-long RNA waiting for the missing UTP (see the ‘Materials and Methods’ section).

We first performed experiments using a DNA template containing the GQ forming sequence in the exon region of PAX9 gene. After immobilizing the stalled elongation complexes on a quartz surface using the biotin–streptavidin interaction (Figure 1B), and washing out unimmobilized elongation complexes three times with 90 μl of an imaging buffer, we resumed transcription elongation by injecting all rNTPs while monitoring single-molecule fluorescence signals using a TIRF microscope (Figure 1B). From a very small number of molecules (0.26%, 6 out of 2285), we could observe FRET change from the low FRET state with a peak at *E* = 0.13 to the high FRET state with a peak at *E* = 0.82 (Figure 1C and Supplementary Figure S1). Interestingly, all of the FRET transitions went through the middle FRET state with a peak at *E* = 0.37 before the transition to the high FRET state. As a trial to identify the conformations corresponding to the middle and high FRET states, we compared their FRET distributions with those of unstructured ssDNA composed of poly-T, and artificially formed GQ of PAX9 gene in the middle of single-stranded region (Figure 1D). The FRET distribution of the high FRET state was indistinguishable from that of GQ. The FRET distribution of the middle FRET state (named as an intermediate state, or I-state hereafter), however, was clearly different from that of the unstructured ssDNA. Experiments with GQ forming sequences from human telomere, MYC and KIT genes revealed that the low GQ forming efficiency in the single-round transcription and the existence of I-state as an intermediate state to GQ formation are general (Supplementary Figure S2). The conformational identity of the I-state is unclear yet, and will be pursued in future studies.

### GQ accumulates under a multiple-round transcription condition

**Figure 2.**
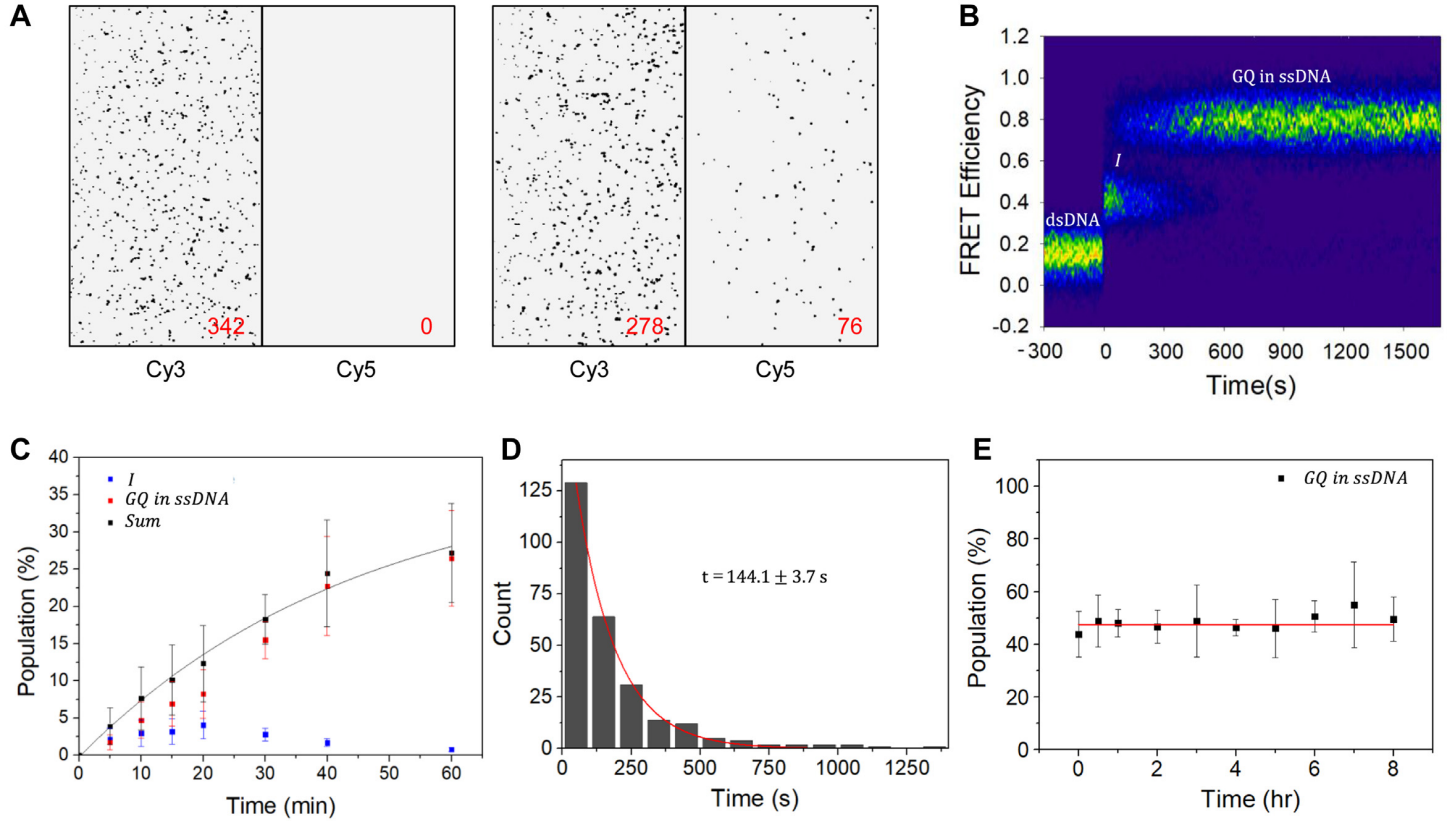
GQ accumulation under a multiple-round transcription condition. (**A**) Cy3 and Cy5 images at Cy3 excitation before transcription start (left) and 1 h after transcription start (right). The numbers of spots are presented in the figure. For better contrast of images, data below a threshold were set to zero. (**B**) A contour plot of FRET efficiency trajectories exhibiting GQ formation. FRET time traces were post-synchronized at first FRET transition events. To correct the photobleaching effect, the decreasing molecule numbers were normalized. (**C**) Time-dependent change of relative populations of I-state (navy) and GQ state (red) with respect to total FRET pairs after transcription start. The population sum of I-sate and GQ state (black) is fitted to a single-exponential function with a time constant of 45.3 ± 5.6 min (black lines). (**D**) A dwell time histogram of I-state. The histogram is fitted to a single-exponential function with a time constant of 144.1 ± 3.7 s (red lines). (**E**) Stability of the cotranscriptionally formed GQ. Time dependence of preformed GQ population was studied by using time-lapse FRET experiments after stopping transcription by washing out RNA polymerase and rNTP. The red line is added as an eye guide. The errors in (**C**) and (**E**) are the standard deviation of the mean of more than three independent experiments.

In the single-round transcription condition described above, GQ formation efficiency was very low. *In vivo*, many genes are transcribed multiple times in a short period of time once activated (22,23). To emulate GQ formation in the actively transcribed genes, we immobilized bare dsDNAs containing the PAX9 GQ forming sequence on a quartz slide and injected T7 RNA polymerases together with rNTPs so that multiple rounds of transcription on the same DNA substrates can occur. As expected, all dsDNA substrates exhibited low FRET efficiency before the injection of RNA polymerase and rNTPs, and no bright spot was observed in the acceptor channel (Figure 2A, left). On the other hand, several bright spots appeared on the acceptor channel 1 h after the injection (Figure 2A, right), indicating efficient formation of GQ structures.

With the increased GQ formation efficiency in the multiple-round transcription condition, we could easily collect time traces exhibiting GQ formation. From 261 such molecules, contour plot of FRET efficiency trajectories was generated by post-synchronizing traces to the first FRET transition events (Figure 2B). The existence of the I-state as an intermediate state in GQ formation pathway is clear. The FRET efficiency of GQ was the same as that presented in Figure 1D, indicating that DNA GQ embedded in the non-template ssDNA strand is formed in the multiple-round transcription as in the single-round transcription. When we introduced G-to-C mutations in the GQ forming sequence, the formation of GQ (the high FRET state) was greatly hindered (Supplementary Figure S3). The transient nature of the I-state and the accumulation of GQ in time were also confirmed by time-lapse single-molecule FRET experiments (Figure 2C); whereas relative population of GQ state monotonically increased, that of the I-state exhibited the falling phase that follows the initial rising phase. The sum of the two populations was nicely fitted to a single-exponential function with a time constant of 45.3 ± 5.6 min. GQ formation efficiency under the multiple-round transcription condition for 1 h was measured as 26.4% (Figure 2C), whereas that of single-round transcription was 0.26%. From the information, we can estimate that in our experimental condition roughly 100 rounds of transcription occurred for 1 h per DNA, which is a bit larger than the number observed *in vivo* (22). Therefore, the GQ formation efficiency observed in Figure 2C may be an overestimation, but this does not invalidate the conclusion that the GQ can accumulate in the actively transcribed genes *in vivo*.

The dwell time histogram of the I-state was well fitted to the single-exponential function with a time constant *τ* of 144.1 ± 3.7 s (Figure 2D). Once the I-state is formed, most of molecules (95%) made a transition to the GQ form except the minor cases (5%) that returned to the low FRET state, or dsDNA. When we consider that the transitions to dsDNA and GQ from the I-state are branched reactions, the apparent transition rate of the I-state (*k* = 1/*τ* = 6.9 × 10^−3^ ± 1.8 × 10^−4^ s^−1^) is given by the sum of the transition rates from I-state to dsDNA (*k*_1_) and to GQ (*k*_2_). On the other hand, the GQ formation probability is given by *k*_2_/(*k*_1_*+ k*_2_). From these considerations, *k*_1_ and *k*_2_ are estimated as 3.4 × 10^−4^ ± 8.0 × 10^−6^ and 6.6 × 10^−3^ ± 1.6 × 10^−4^ s^−1^, respectively. To check the stability of cotranscriptionally formed GQ, we accumulated the GQ form by allowing the multiple rounds of transcription to occur for 90 min, and then monitored how the GQ population changes as a function of time after washing out RNA polymerases and rNTP from the reaction chamber. A change of the GQ population was not detectable for 8 h (Figure 2E), confirming the extreme stability of the cotranscriptionally formed GQ structure by itself. Experiments with other GQ forming sequences revealed that the observations above (the accumulation of GQ in the multiple-round transcription condition and the high stability of GQ once it is formed) are general (Supplementary Figure S4) even though their actual GQ formation efficiencies vary.

GQ is known to have several conformational isomers. The improved GQ forming efficiency under the multiple-round transcription condition allowed us to identify the isomeric form of the cotranscriptionally formed GQs using the chemical ligands that specifically bind to either the parallel form (*N*-methyl mesoporphyrin IX) or the antiparallel form (crystal violet). We found that all tested GQs are dominantly in the parallel form (Supplementary Figure S5) like the GQ artificially formed (20).

### Cotranscriptionally formed R-loop facilitates GQ formation

**Figure 3.**
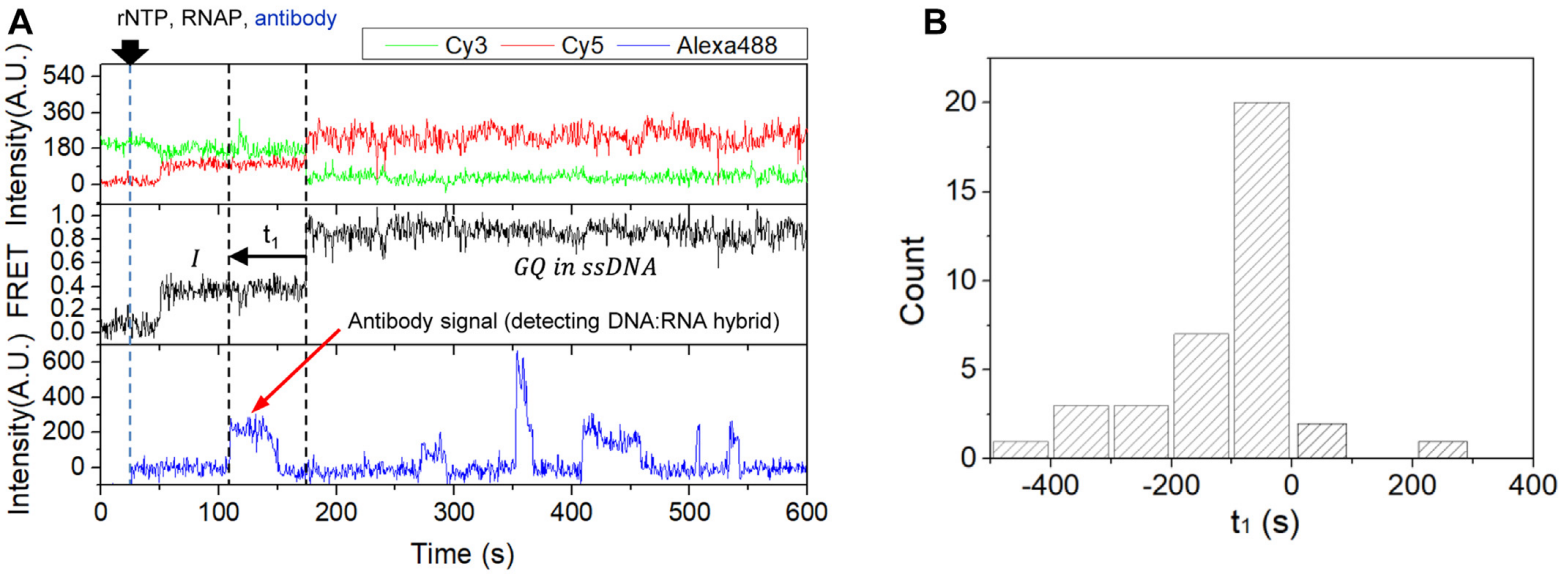
GQ formation facilitated by R-loop. (**A**) Representative time traces showing R-loop formation prior to GQ formation: Cy3 (top, green) and Cy5 (top, red) fluorescence intensities at Cy3 excitation, the corresponding FRET (middle) and Alexa488 fluorescence intensity at Alexa488 excitation (bottom). (**B**) A histogram of the time difference between GQ formation and antibody binding [*t*_1_ as defined in the middle panel of (**A**)]. Most of molecules showed antibody binding in the I-state prior to the GQ formation.

To determine the temporal order of GQ formation and the R-loop formation during transcription, we prepared Alexa488-labeled antibody S9.6 (see the ‘Materials and Methods’ section), which is known to bind to DNA:RNA hybrid with a high specificity (24). When we injected the Alexa488-labeled antibody S9.6 (33 nM) together with RNA polymerase and rNTPs into the channel where dsDNA containing the PAX9 GQ forming sequence is immobilized, representative fluorescence time traces in Figure 3A show that the antibody binding occurred before GQ formation, which is generally observed in most molecules exhibiting the GQ formation (Figure 3B). We confirmed that antibody S9.6 specifically bound to DNA:RNA hybrid and the nonspecific binding of the antibody to dsDNA was negligible, and the antibody does not promote the formation of the R-loop (Supplementary Figure S6A–D). In the experimental condition, the association time of the antibody to DNA:RNA hybrid was measured as 24.8 ± 0.88 s on average (Supplementary Figure S6E).

### GQ remains stable after R-loop degradation

**Figure 4.**
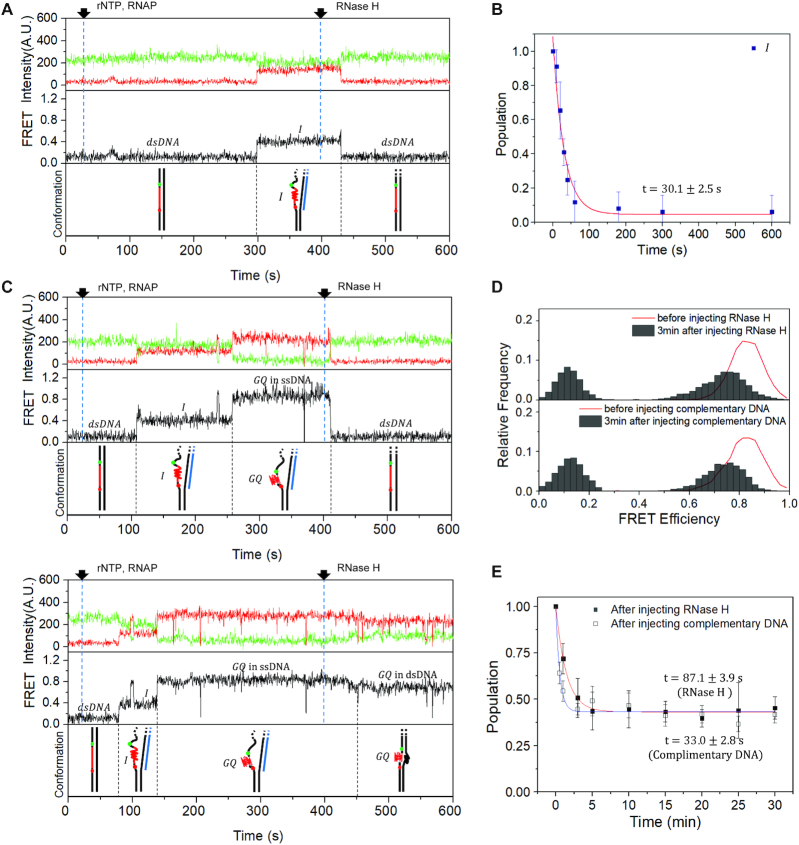
Persistence of GQ after R-loop degradation. (**A**) Representative time traces of Cy3 (top, green) and Cy5 (top, red) fluorescence intensities at Cy3 excitation, the corresponding FRET (middle) and the corresponding conformations of nucleic acids (bottom) that show a FRET transition from I-state to dsDNA after RNase H treatment. A mixture of RNA polymerase and rNTP was injected at 25 s to resume the elongation, and RNase H was added at 400 s to degrade R-loops. (**B**) Lifetime of I-state after RNase H treatment. The histogram is fitted to a single-exponential function with a time constant of 30.1 ± 2.5 s (red lines). (**C**) Representative time traces exhibiting FRET transitions from GQ to dsDNA (top) and to a new high FRET state (middle), and the corresponding conformations of nucleic acids (bottom). The same color convention is used as in (**A**). (**D**) Comparison of FRET histograms of the cotranscriptionally formed GQ after RNase treatment (top), and after the injection of the ssDNA (1 μM, bottom) complementary to the GQ forming sequence (Supplementary Table S1). The FRET histogram of GQ before the treatments (red lines) is shown as an eye guide. (**E**) The relative populations of GQ remaining after the RNase H treatment (solid squares) and ssDNA treatment (open squares). The data are fitted to single-exponential functions with time constants of 87.1 ± 3.9 s (red lines) and 33.0 ± 2.8 s (blue lines), respectively.

Figure 3 shows that the R-loop formation occurs prior to and facilitates the GQ formation. To study how R-loop affects the GQ stability once it is formed, we degraded R-loop by treating RNase H (New England Biolabs) a few minutes after the start of a multiple-round transcription using a DNA template containing the PAX9 GQ forming sequence. We found that the I-state is efficiently transformed into the duplex form in the presence of RNase H (Figure 4A) with a time constant of 30.1 ± 2.5 s (Figure 4B). On the other hand, we observed that there are two types of transitions from the GQ state: the one that rapidly decays to dsDNA (Figure 4C, top), and the other that makes a transition to the new high FRET state with a peak at *E* = 0.75 (Figure 4C, bottom). The FRET histogram of the new high FRET state was similar to the FRET histogram observed when a complementary ssDNA was injected to GQ (Figure 4D) and the FRET histogram obtained from the artificially formed GQ in the crowding condition (Supplementary Figure S7). This observation generally valid for all tested GQ forming sequences (Supplementary Figure S7) indicates that the new high FRET state corresponds to GQ embedded in dsDNA, whereas the original high FRET state corresponds to GQ embedded in ssDNA (Figure 1D). This observation also indicates that there are at least two GQ structures with different levels of resistance to the RNase H treatment. The existence of the GQ structure resistant to the RNase H treatment (43.3%) is also clear in Figure 4E (solid squares) that shows GQ population as a function of time after the RNase treatment. The similar amount of GQ was observed to remain after the injection of an ssDNA complementary to GQ forming sequence (Figure 4E, open squares), indicating that the existence of the ultrastable GQ is the intrinsic property of GQ forming sequence of PAX9 gene. GQ forming sequences from other genes exhibited varying degrees of resistance to the RNase H treatment (Supplementary Figure S8A). Interestingly, we found that the resistance to the RNase H treatment decreases with increasing loop length of the GQ forming sequence (Supplementary Figure S8B).

### GQ on the non-template strand promotes the R-loop formation during transcription

**Figure 5.**
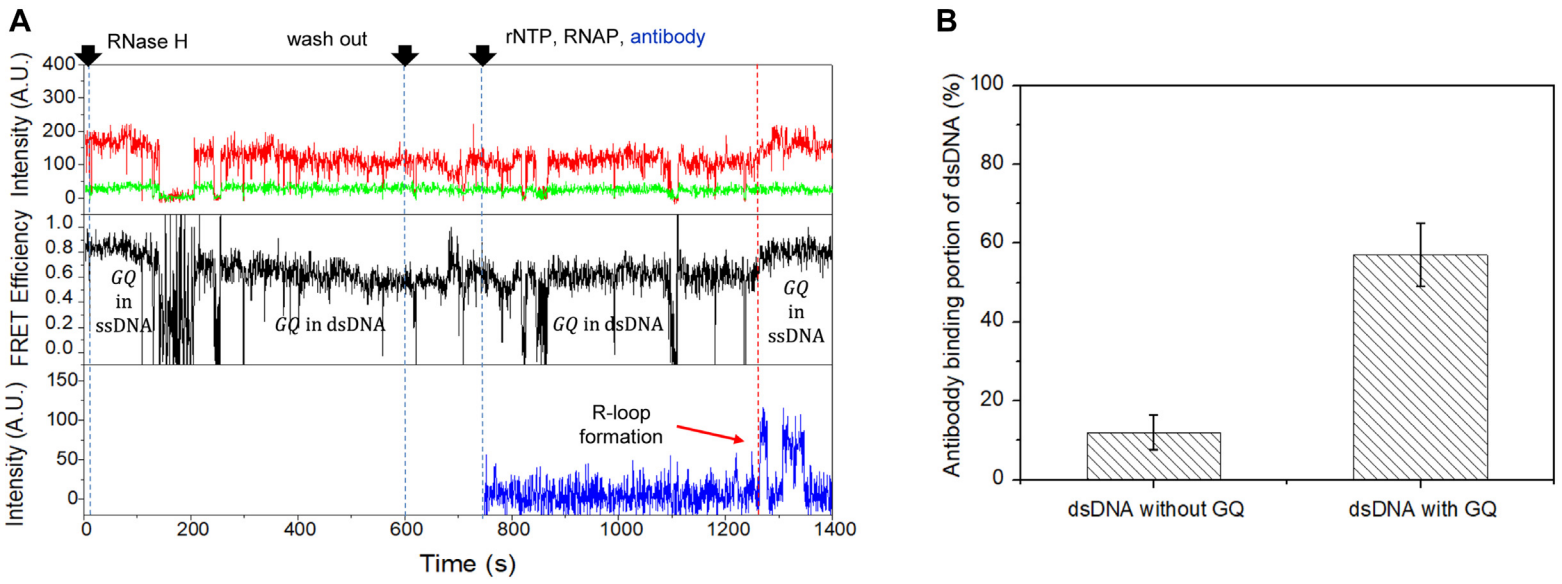
R-loop formation facilitated by GQ. (**A**) Representative time traces showing R-loop formation during transcription on dsDNA with GQ on the non-template strand: Cy3 (top, green) and Cy5 (top, red) fluorescence intensities at Cy3 excitation, the corresponding FRET (middle) and Alexa488 fluorescence intensity at Alexa488 excitation (bottom). It is noticeable that the FRET efficiency of GQ decreases after R-loop degradation by RNase H, and recovers the original value after R-loop formation. (**B**) The portion of dsDNA that exhibited antibody binding for 20 min after the start of the multiple-round transcription. The antibody binding (R-loop formation) probability significantly increases when dsDNA has GQ on the non-template strand.

Finally, we tested how GQ existing in the non-template strand affects the next round of transcription and R-loop formation. We first prepared GQ-containing dsDNAs by accumulating PAX9 GQ under the multiple-round transcription condition for 40 min, and then degrading R-loops using RNase H. After washing the reaction chamber three times using 90 μl of the imaging buffer, we injected rNTP, T7 RNA polymerase and Alexa488-labeled antibody S9.6 to restart multiple rounds of transcription and monitor the formation of R-loops. A few minutes after the injection, R-loop was re-formed and non-template strand containing GQ transited from dsDNA to ssDNA (red dashed line, Figure 5A). Compared to dsDNA without GQ, the antibody binding portion of dsDNA with GQ increased significantly (Figure 5B), indicating that GQ in the non-template strand promotes the R-loop formation during transcription. When we repeat the experiment using GQ prepared in the crowding condition, we observed the same effect (Supplementary Figure S9).

## DISCUSSION

**Figure 6.**
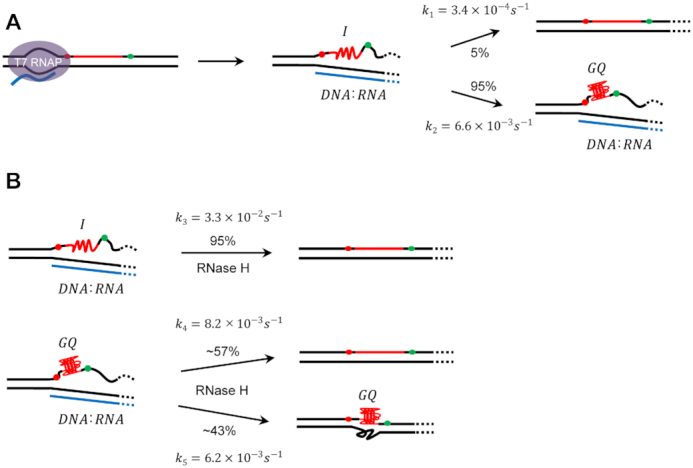
Models for cotranscriptional formations of GQs and R-loops. (**A**) The R-loop formation occurs prior to the GQ formation. Once R-loop is formed, the exposed non-template strand makes the I-state. Most of molecules (95%) in the I-state make transitions to GQ state with a rate constant of 6.6 × 10^−3^ ± 1.6 × 10^−4^ s^−1^ except the rare cases (5%) that return to dsDNA with a rate constant of 3.4 × 10^−4^ ± 8.0 × 10^−6^ s^−1^. (**B**) After R-loop is degraded, most of molecules (95%) in the I-state return to dsDNA. On the other hand, significant portion of molecules in the GQ state (43%) survives the R-loop degradation. The reaction rates in the figure are the values obtained using 17 U/ml of RNase H.

It has been long known that GQ and R-loop formations during transcription are coupled, but their exact coupling mechanism was not clear. Two different scenarios can be imagined. It is possible that GQ is formed first, and the resulting long single-stranded region on the template facilitates the R-loop formation. On the other hand, R-loop can form first, and the resulting long single-stranded region on the non-template strand facilitates GQ formation. We developed single-molecule fluorescence assays to monitor the cotranscriptional formation of GQ and R-loop in real time, and resolved the issue by clearly demonstrating that R-loop forms prior to GQ (Figure 6A).

We discovered that the I-state exists as an intermediate state in the GQ formation pathway. Even though most of molecules in the I-state eventually make a transition to the GQ form (Figure 6A), the presence of the I-state may provide a time window for the cell to resolve the R-loop and recover the intact dsDNA with the help of protein factors such as RNase H (Figure 6B).

The GQ formation efficiency per single-round transcription was extremely low. Once formed, however, some GQs were ultrastable; even the treatment of RNase H could not fully recover the intact dsDNA. As a consequence, GQ efficiently accumulated in the actively transcribed genes. We also found that GQs in the non-template strand facilitate the R-loop formation during next rounds of transcription, revealing the existence of a positive feedback mechanism of GQ and R-loop formations. *In vivo*, there exist many helicases and ssDNA binding proteins that unfold GQs and R-loops. To make a realistic picture of what happens *in vivo*, therefore, the dynamical actions of these proteins should also be incorporated. However, the resolution of either GQ or R-loop by these proteins is not enough, but both of them should be resolved to stop the accumulation of GQ and R-loop. R-loop is often called a double-edged sword. When it is formed in an unscheduled fashion, and not properly resolved, R-loop is known to cause genome instability. Thus, the existence of a positive feedback loop of GQ and R-loop formations can exacerbate the threat of DNA damage and genome instability in vulnerable genes. On the other hand, R-loops formed near transcription start and termination sites play positive biological roles in gene regulation. In this case, the existence of the positive feedback loop can contribute to timely formation of the R-loops.

One of the intriguing questions we could not address in this paper is how the interaction of an R-loop with an RNA polymerase of the next-round transcription is controlled. R-loops may block the elongation as previously reported (25), or promote it as recently reported (26). Further studies are required to answer the question. Our study used T7 RNA polymerase. In prokaryotes and eukaryotes, however, it is possible that GQ and R-loop formations are differently related due to RNA polymerase-specific effects. Further studies using bacterial and eukaryotic RNA polymerases are required. In this study, we could not determine the structural identity of the I-state. Considering the importance of the I-state in GQ formation and dsDNA recovery, we need to clarify the structure of the I-state. We showed that a parallel GQ is formed in the PAX9 gene during transcription. Intriguingly, however, the treatment of RNase H and the injection of ssDNA complementary to the GQ forming sequence indicate that there exists a structural heterogeneity in the parallel form of GQ. The exact nature of the heterogeneity needs to be clarified.

## Supplementary Material

gkaa695_Supplemental_File
