# Supplementary material for: Single-molecule fluorescence studies on cotranscriptional G-quadruplex formation coupled with R-loop formation
Source: Nucleic Acids Res. 2020 Aug 18;48(16):9195–203. doi: 10.1093/nar/gkaa695 (PMC7498336; doi:10.1093/nar/gkaa695)
Supplement: gkaa695_Supplemental_File [file gkaa695_supplemental_file.pdf]

**Supplementary Data**

# Single-molecule fluorescence studies on cotranscriptional G-quadruplex formation coupled with R-loop formation

## Contents


**Supplementary Table S1.** DNA sequences for the experiments.

**Supplementary Figure S1.** Other single-molecule time traces showing GQ formation during single-round transcription.

**Supplementary Figure S2.** Single-round transcription experiments with other GQ forming sequences.

**Supplementary Figure S3.** GQ mutant experiments.

**Supplementary Figure S4.** Multiple round transcription on other GQ forming sequence.

**Supplementary Figure S5.** Identification of GQ isomers.

**Supplementary Figure S6.** Characterization of antibody S9.6.

**Supplementary Figure S7.** Comparison of FRET histograms of the cotranscriptionally formed GQ after RNase H treatment and those of GQ formed in the crowding condition.

**Supplementary Figure S8.** Resistances to the RNase H treatment.

**Supplementary Figure S9.** Effect of GQ in non-template strand on the next round of transcription and R-loop formation.

**Supplementary Table S1.** DNA sequences for the experiments.

| Sample name | | Sequence (5' to 3') |
|---|---|---|
| PAX9 | Non-template | ATCAGGTCTAATACGACTCACTATAGGAAGAGAAAGT/iCy5/TTCTGGGAGGGAGGGAGGGTGTA/iCy3/CTGATGCGTTCCACTCGC |
| | Template | GCGAGTGGAACGCATCAGTACACCCTCCCTCCCTCCCAGAAACTTTCTCTTCCTATAGTGAGTCGTATTAGACCTGAT/biotin/ |
| | Complimentary | GCGAGTGGAACGCATCAGTACACCCTCCCTCCCTCCCAGAAACTTTCTCTTCC |
| Human telomere | Non-template | ATCAGGTCTAATACGACTCACTATAGGAAGAGAAAGT/iCy5/TTCTGGGTTAGGGTTAGGGTTAGGGTGTA/iCy3/CTGATGCGTTCCACTCGC |
| | Template | GCGAGTGGAACGCATCAGTACACCCTAACCCTAACCCTAACCCAGAAACTTTCTCTTCCTATAGTGAGTCGTATTAGACCTGAT/biotin/ |
| | Complimentary | GCGAGTGGAACGCATCAGTACACCCTAACCCTAACCCTAACCCAGAAACTTTCTCTTCC |
| MYC | Non-template | ATCAGGTCTAATACGACTCACTATAGGAAGAGAAAGT/iCy5/TTCTGGGTGGGTAGGGTGGGTGTA/iCy3/CTGATGCGTTCCACTCGC |
| | Template | GCGAGTGGAACGCATCAGTACACCCACCCTACCCACCCAGAAACTTTCTCTTCCTATAGTGAGTCGTATTAGACCTGAT/biotin/ |
| | Complimentary | GCGAGTGGAACGCATCAGTACACCCACCCTACCCACCCAGAAACTTTCTCTTCC |
| KIT | Non-template | ATCAGGTCTAATACGACTCACTATAGGAAGAGAAAGT/iCy5/TTCTGGGAGGGCGCTGGGAGGAGGGTGTA/iCy3/CTGATGCGTTCCACTCGC |
| | Template | GCGAGTGGAACGCATCAGTACACCCTCCTCCCAGCGCCCTCCCAGAAACTTTCTCTTCCTATAGTGAGTCGTATTAGACCTGAT/biotin/ |
| | Complimentary | GCGAGTGGAACGCATCAGTACACCCTCCTCCCAGCGCCCTCCCAGAAACTTTCTCTTCC |
| Poly-T | Non-template | ATCAGGTCTAATACGACTCACTATATTTTTTTTTTT/iCY5/TTTTTTTTTTTTTTTTTTTTTTT/iCy3/T |
| Template up to promoter | | TATAGTGAGTCGTATTAGACCTGAT/biotin/ |

- promoter sequence is underlined
- red sequence : GQ forming sequence
- complimentary sequence was used to mimic RNase H experiment
- template up to promoter was used to expose GQ forming strand as a ssDNA form from +1 site

**Supplementary Figure S1.** Other single-molecule time traces showing GQ formation during single-round transcription.

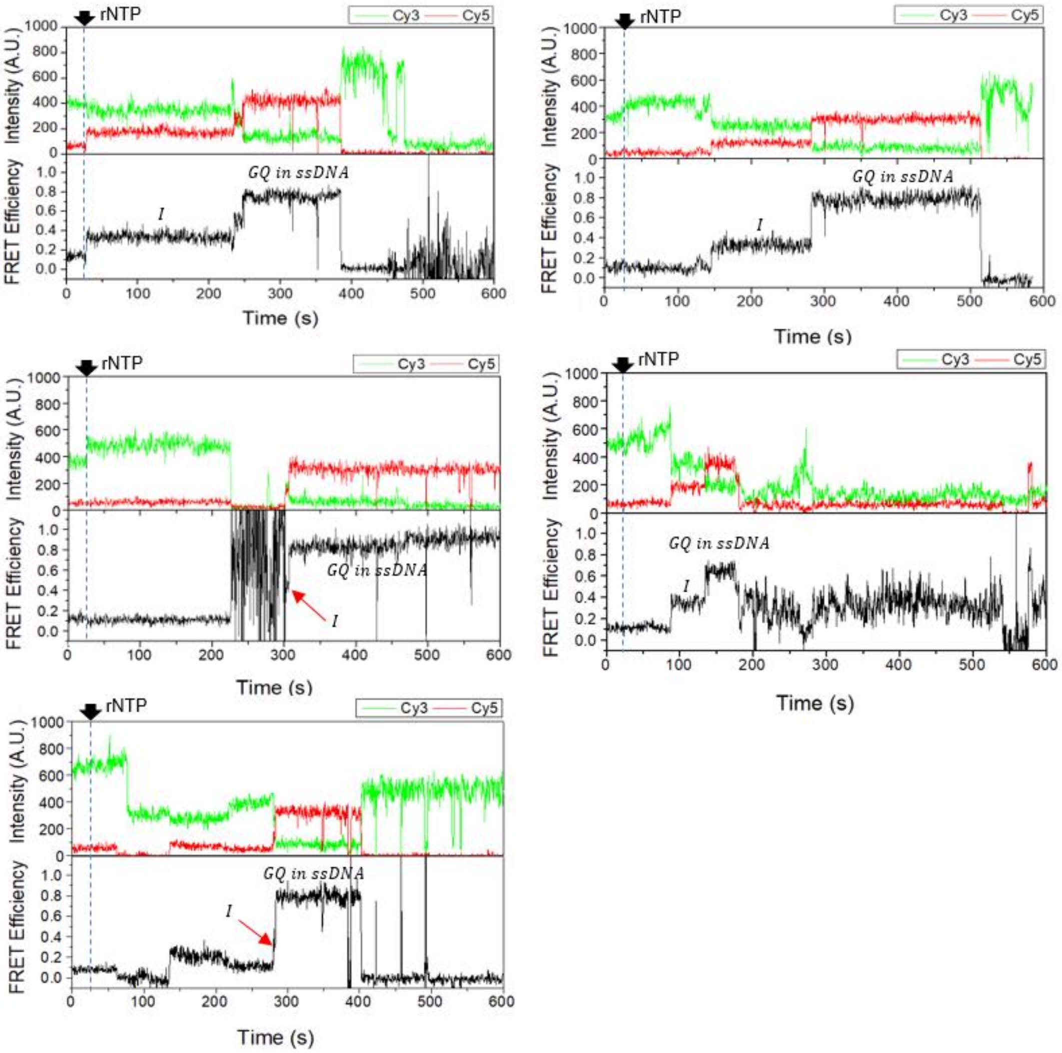

**Supplementary Figure S2.** Single-round transcription experiments with other GQ forming sequences.

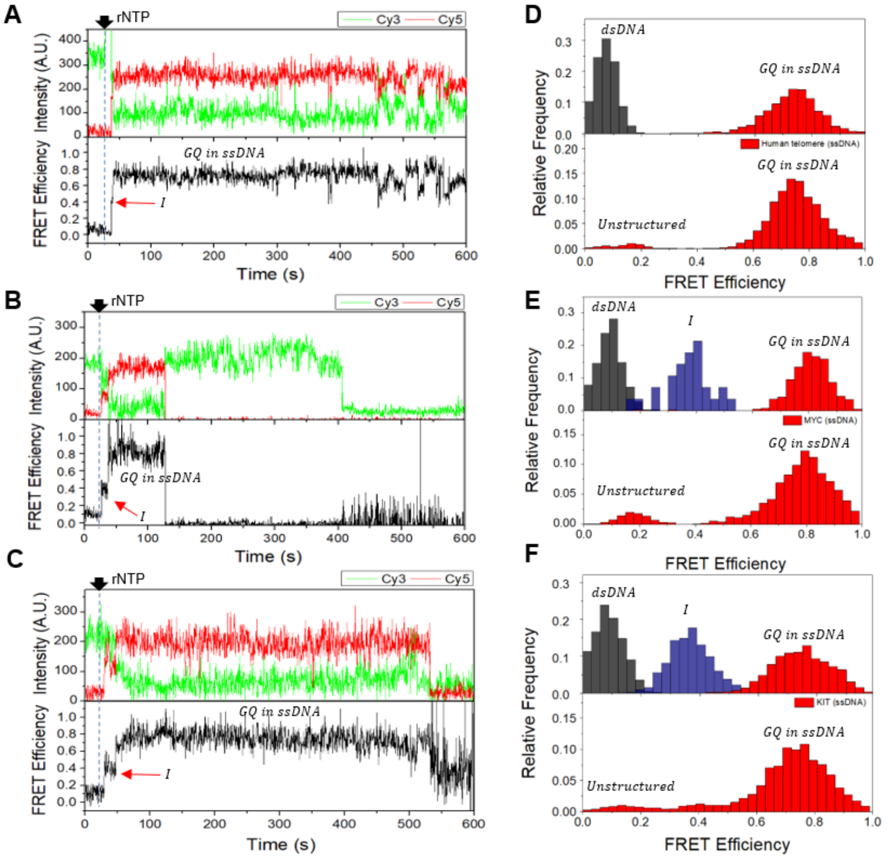


Representative time traces of Cy3 (top, green) and Cy5 (top, red) fluorescence intensities at Cy3 excitation, and the corresponding FRET (bottom) that show GQ formation of GQ forming sequences from human telomere (A), MYC (B), and KIT (C) genes. (D) FRET histograms of the low FRET state (top, black), middle FRET state (top, navy), and high FRET state (top, red) are compared with the FRET histograms of non-template ssDNA containing the GQ forming sequences from human telomere (D), MYC (E), and KIT (F) genes. In case of human telomere, the I-state was too brief to make its histogram.

**Supplementary Figure S3.** GQ mutant experiments.

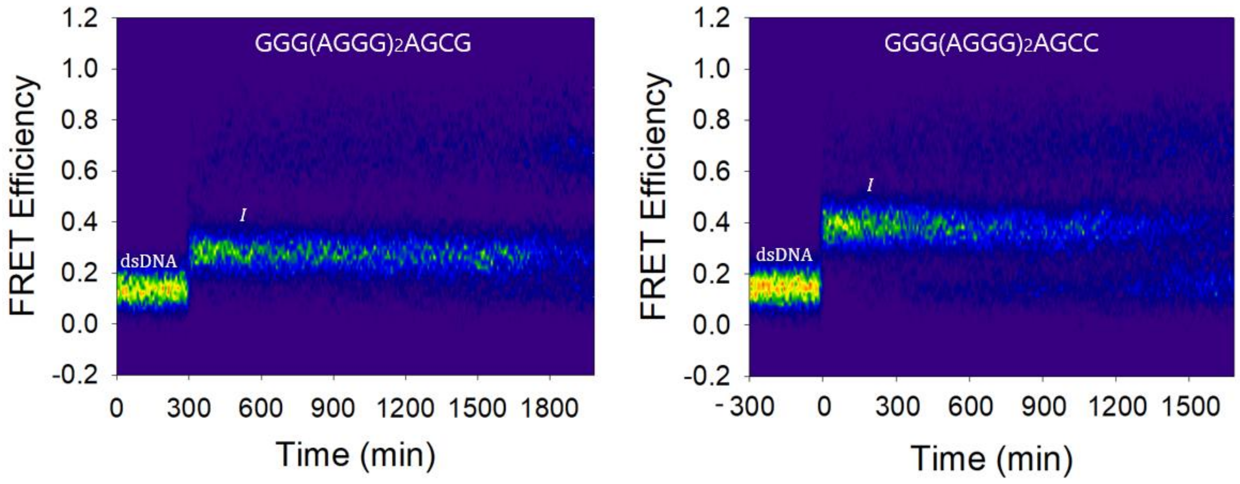


Contour plots of FRET efficiency trajectories exhibiting FRET changes after the start of multiple-round transcription. It is clear that GQ formation (a transition to the high FRET state) is significantly hindered in the mutant samples.

**Supplementary Figure S4.** Multiple round transcription on other GQ forming sequence.

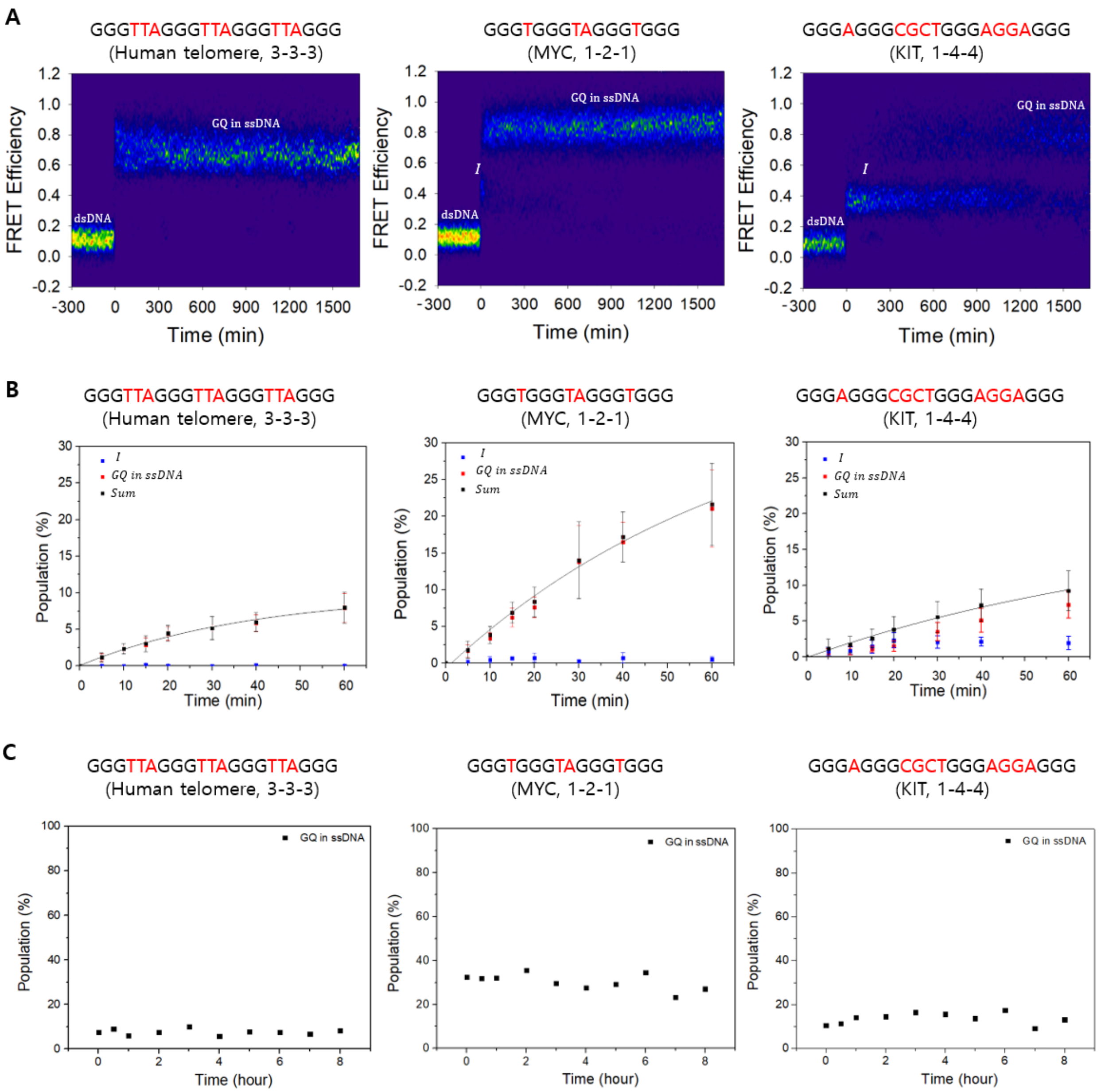


(A) Contour plots of FRET efficiency trajectories showing GQ accumulation after the start of multiple-round transcription for other GQ forming sequences. (B) Time-dependent change of relative populations of I-state (navy) and GQ state (red) with respect to total FRET pairs after transcription start. The population sum of I-sate and GQ state (black) is fitted to a single-exponential function with a time constant of 39, 68, 86 min, respectively (black lines). (C) Stability of the cotranscriptionally formed GQ. Time-dependency of pre-formed GQ population was studied by using time-lapse FRET experiments after stopping transcription by washing out RNA polymerase and rNTP. GQ populations do not show noticeable changes for 8 hours.

**Supplementary Figure S5.** Identification of GQ isomers.

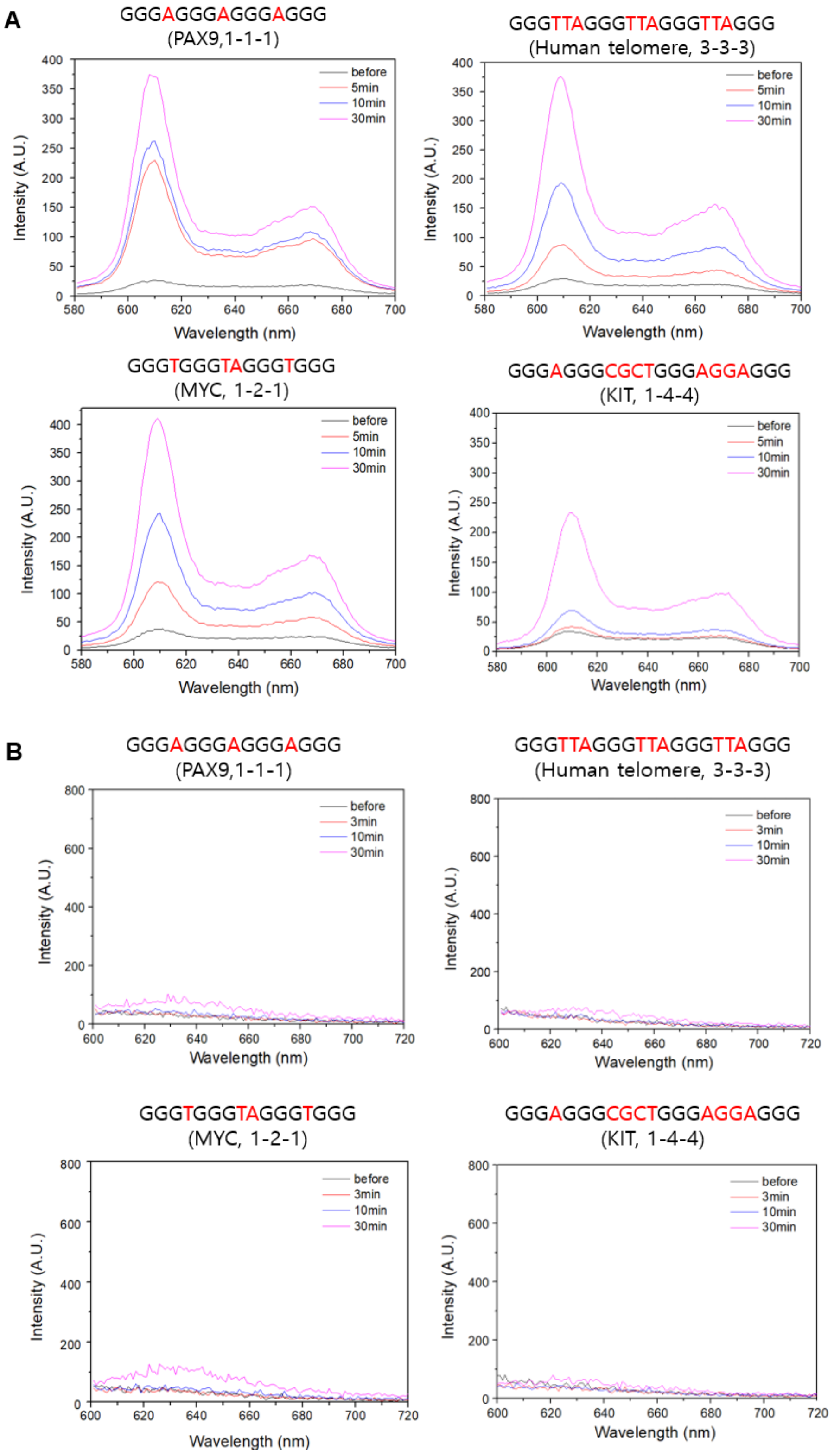

(A) Emission spectra of NMM before and a few minutes after the transcription start. Transcription was triggered by injecting RNA polymerase (20 nM) into the solution containing NMM (1 uM), double-stranded DNA (100 nM) and rNTP (2 mM). The intensity of NMM fluorescence increased over time. (B) When CV (10 uM) was used instead of NMM, there was no significant change in the fluorescence intensity.

**Supplementary Figure S6.** Characterization of antibody S9.6.

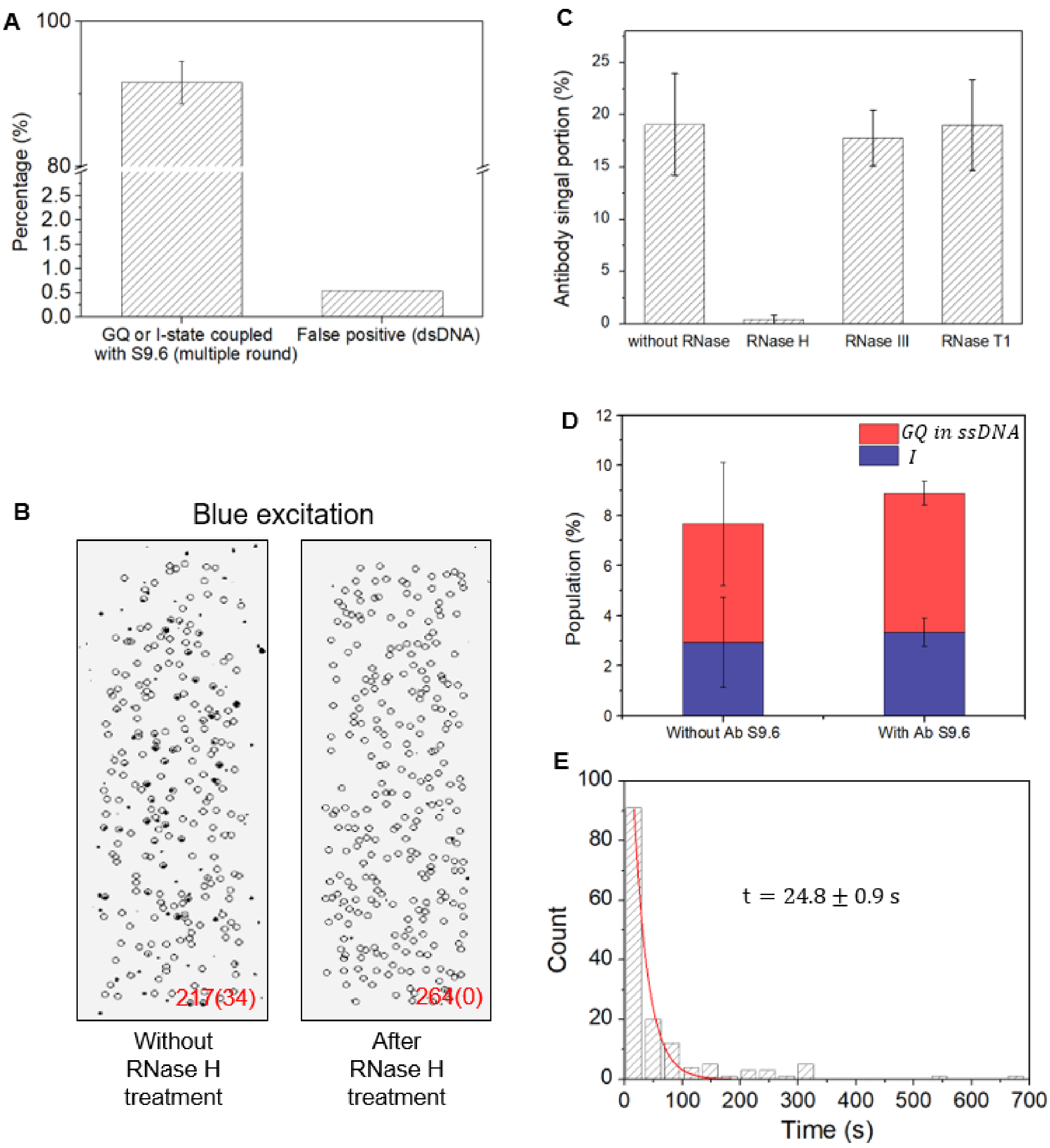

(A) When fluorescently-labeled antibody S9.6 was injected together with RNA polymerase and rNTP, 91.6% of molecules with a FRET change either to the I-state or GQ exhibited the antibody biding. The observation was made for 30 min. On the other hand, when the experiment was performed without RNA polymerase and rNTP, only 0.5% of dsDNA exhibited the antibody binding. (B) (left) To further show that antibody S9.6 specifically binds to DNA:RNA hybrid in our experimental condition, we preformed R-loops by performing the multiple round transcription for 40 min, and injected antibody S9.6. It is clear that antibody binding (34, black) is well co-localized with dsDNA (217, open circles). (right) When R-loops were treated with RNase H for 10 min before the antibody injection, no antibody binding was observed. (C) Antibody binding portion after 40 min of multiple round transcription. Antibody was injected 10 min after the treatment of RNase H, RNase III or RNase T1 treatment, or without RNase treatment. (D) The multiple round transcription was performed for 10 min with and without S9.6 (33 nM), and the total number of molecules in either the I-state and GQ were counted. No apparent change was observed, indicating that antibody S9.6 does not help R-loop formation. All experiments were performed with dsDNA containing PAX9 GQ forming sequence. (E) To measure association time of antibody S9.6 for the DNA:RNA hybrid, we preformed DNA:RNA hybrid by incubating dsDNA with RNA polymerase and rNTP for 1 hr, and injected the fluorescently-labeled antibody S9.6 (33 nM) to the channel. The association time histogram of the antibody was nicely fitted to a single-exponential function with a time constant of 24.8 s (red lines).

**Supplementary Figure S7.** Comparison of FRET histograms of the cotranscriptionally formed GQ after RNase H treatment and those of GQ formed in the crowding condition.

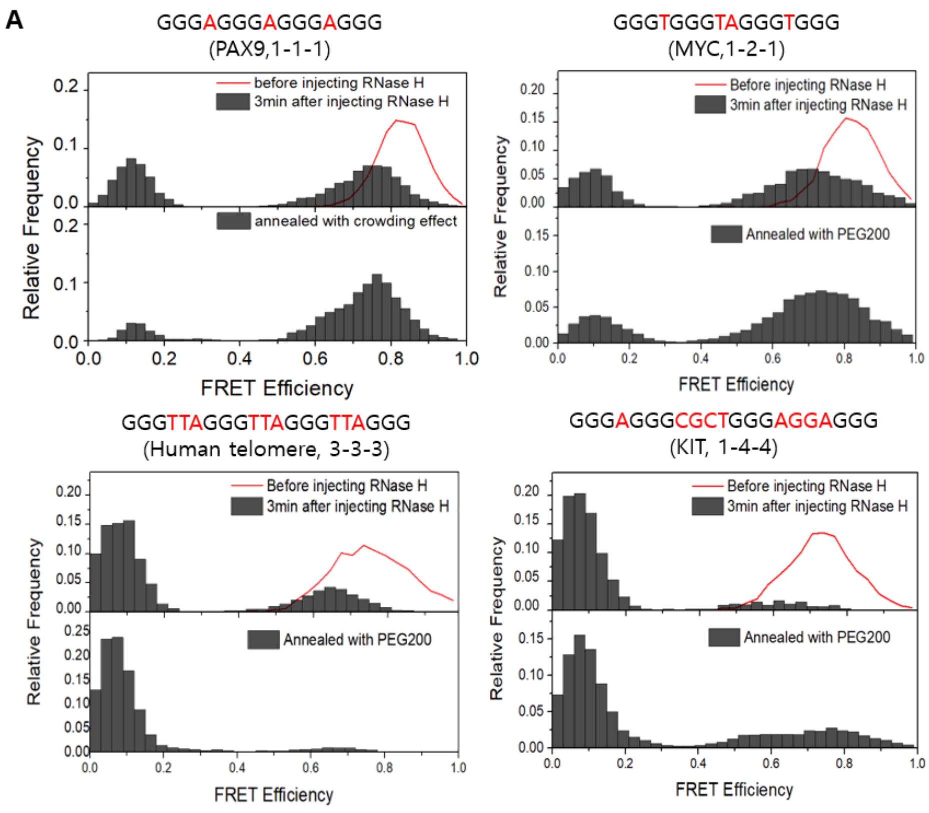


For all tested GQ forming sequences, the FRET histograms of the cotranscriptionally formed GQ after RNase H treatment (top panels) are similar to those of GQ artificially formed in the crowding condition (bottom panels). The FRET histograms of the cotranscriptionally formed GQ before RNase H treatment (red lines) are shown as an eye guide.

**Supplementary Figure S8.** Resistances to the RNase H treatment.

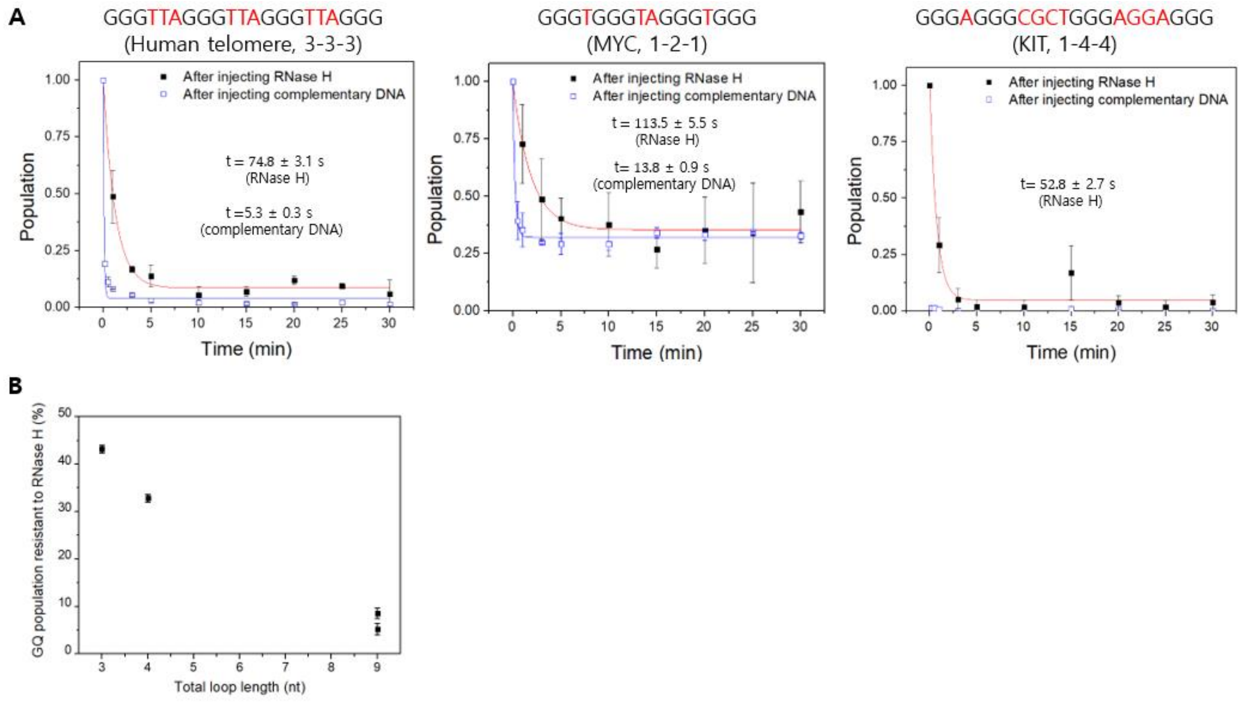


(A) The relative populations of GQ remaining after the RNase H treatment (solid squares), and single-stranded DNA treatment (open squares). The data are fitted to single-exponential functions. Time constants were presented in the Figures. (B) A correlated plot of the GQ population resistant to the RNase H treatment and the total loop length of the four different GQ forming sequences.

**Supplementary Figure S9.** Effect of GQ in non-template strand on the next round of transcription and R-loop formation.

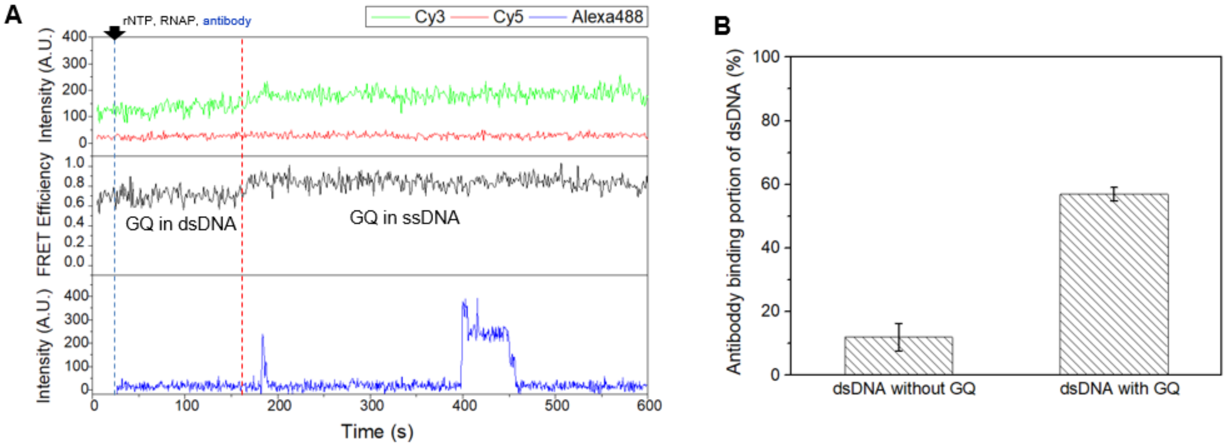


(A) Representative time traces showing R-loop formation during transcription on dsDNA with GQ on the non-template strand: Cy3 (top, green) and Cy5 (top, red) fluorescence intensities at Cy3 excitation, the corresponding FRET (middle), and Alexa488 fluorescence intensity at Alexa488 excitation (bottom). dsDNA containing PAX9 GQ on non-template strand was prepared using the crowding effect (Materials and Methods). After transcription initiation, GQ in dsDNA was exposed as a ssDNA form and antibody signal was coupled. (B) The portion of dsDNA that exhibited antibody binding for 20 min after the start of the multiple round transcription. The antibody binding (R-loop formation) probability significantly increases when dsDNA has GQ on the non-template strand.
